# Effects of High-Intensity Intermittent Training Combined with *Asparagus officinalis* Extract Supplementation on Cardiovascular and Pulmonary Function Parameters in Obese and Overweight Individuals: A Randomized Control Trial

**DOI:** 10.3390/jfmk10020202

**Published:** 2025-06-01

**Authors:** Tadsawiya Padkao, Piyapong Prasertsri

**Affiliations:** Faculty of Allied Health Sciences, Burapha University, Chonburi 20131, Thailand; tadsawiya@go.buu.ac.th

**Keywords:** obesity, asparagus, high-intensity intermittent training, blood pressure, pulmonary function, endothelial function, heart rate variability

## Abstract

**Background:** High-intensity intermittent training (HIIT) has been proven to improve cardio-metabolic and respiratory health outcomes. In addition, 20-hydroxyecdysone from plant extracts has been studied for its anabolic effects. However, studies examining these two interventions in individuals who are obese or overweight are limited. This study, thus, examined the effects of HIIT combined with *Asparagus officinalis* (*A. officinalis*) extract supplementation on cardiovascular and pulmonary function parameters in obese and overweight individuals. **Methods:** Seventy-two obese and overweight participants were randomized into four groups (*n* = 18 each): the control (CON) group; HIIT group (HIIT for 3 days/week); AOE (*A. officinalis* extract) group (supplementation with 20E at 1.71 mg/kg/day); and HIIT + AOE group. Pre- and 12-week post-intervention measures included heart rate (HR), HR variability, endothelial function, blood pressure (BP), BP variability, pulmonary function and volume, respiratory muscle strength, chest expansion, and body composition. **Results:** The HIIT + AOE group showed better HR variability with higher high-frequency power and a lower low-frequency/high-frequency ratio (both *p* = 0.038) compared to the CON group. The peak blood flow increased in both the HIIT (*p* = 0.03) and HIIT + AOE (*p* = 0.028) groups, but only the HIIT group had a shorter vascular recovery time (*p* = 0.048). The maximum expiratory pressure was increased in both the HIIT and HIIT + AOE groups compared to the CON group (*p* = 0.029 and *p* = 0.041). The ratio of forced expiratory volume in one second to forced vital capacity, the percent-predicted FEV_1_/FVC, and chest wall expansion were higher in the HIIT + AOE group than in the CON group (*p* = 0.047, *p* = 0.038, and *p* = 0.001). The waist-to-hip ratio was lower in the HIIT + AOE group than in the CON group (*p* = 0.043). There were no significant differences in HR, BP, BP variability, or pulmonary volume parameters among groups. **Conclusions:** The combination of HIIT with *A. officinalis* extract supplementation markedly improves HR variability. Moreover, it also greatly improves expiratory muscle strength, chest wall expansion, pulmonary function, and body composition parameters in obese and overweight individuals.

## 1. Introduction

Obesity is a major risk factor for increased all-cause mortality, particularly due to cardiovascular diseases [1]. It is also related to systemic inflammation, cancer, and respiratory comorbidities [2]. In individuals with obesity, an imbalance in autonomic nervous system activity can increase the risk of non-communicable diseases, such as hypertension and type 2 diabetes, and can lead to higher cardiovascular disease mortality [3]. This imbalance can manifest in a change in either heart rate (HR) variability or blood pressure (BP) variability, which predominantly originates from increased sympathetic activity and reduced vagal control [4,5].

Moreover, pulmonary function is frequently significantly altered in obesity. This alteration is a consequence of the reduced compliance of the lungs, chest wall, and entire respiratory system [6,7,8,9,10]. These underlying changes alter the breathing pattern, resulting in a substantial reduction in both the expiratory reserve volume (ERV) and the static resting volume of the lungs, known as the functional residual capacity (FRC) [11]. Dynamic measures of pulmonary function, such as forced vital capacity (FVC) and forced expiratory volume in one second (FEV_1_), are slightly reduced in obesity, but the FEV_1_/FVC ratio is usually unaffected [10,12,13,14,15]. Taken together, these findings suggest that obesity not only disturbs pulmonary function but also significantly diminishes pulmonary volume.

Previous studies have examined non-pharmacologic treatments, including physical activity and exercise, as effective strategies for managing obesity and overweight [16,17]. High-intensity intermittent training (HIIT) is a structured exercise regimen that alternates short bursts of vigorous activity with periods of complete rest or low-intensity recovery [18]. The duration of exercise and rest periods ranges from 6 s to 4 min, with training programs typically lasting between 2 and 15 weeks. [19]. While the positive effects of HIIT on muscular performance, muscle thickness, and cardiopulmonary fitness (e.g., maximal oxygen uptake) are well established [20,21,22], the efficacy of HIIT in improving cardiovascular and pulmonary function indices—such as HR, HR variability, endothelial function, BP, and BP variability—in obese and overweight individuals has not yet been thoroughly examined.

Over the past two decades, one class of dietary supplements that has garnered increasing interest is ecdysteroids, which are found in animals, plants (phytoecdysteroids), and fungi [23]. The most common type of phytoecdysteroid is 20-hydroxyecdysone (20E), which is often the predominant phytoecdysteroid in plants [24]. Asparagus or *Asparagus officinalis* (*A. officinalis)*, also known as the “king of vegetables”, has been identified as a source of 20E, particularly in its hard stem by-product, which contains relatively high levels of 20E, approximately 2.34 mg/g dry weight [25]. Pharmacological studies of 20E in mammals have clearly demonstrated its anabolic properties, as well as its potential benefits in fat reduction, anti-diabetic activity, anti-inflammatory and antioxidant effects, vasorelaxation, cardioprotective properties, and neuromuscular and pulmonary protection [24]. Studies conducted in healthy individuals have demonstrated that 20E enhances physical performance and exhibits anabolic effects in both athletes and young adults [23]. Only two studies have investigated the effects of 20E in individuals with obesity. In the first study, obese participants received 20E at a dose of 2 × 50 mg/day for approximately three months, resulting in reductions in body weight, waist circumference, and body fat; a decrease in systemic inflammation; a 34% reduction in C-reactive protein levels; a 2.9% decrease in total cholesterol and triglycerides; and an increase in muscle strength [26]. In the second study involving obese individuals, the administration of 20E at doses of 100 or 200 mg/day for similar durations demonstrated the potential to reduce body weight, lower cholesterol and C-reactive protein levels, and prevent osteoporosis [27]. These studies revealed that 20E supplementation for approximately three months reduced body weight, waist circumference, body fat, systemic inflammation, and blood lipids; increased muscle strength; and prevented osteoporosis.

Despite existing research, there remains a lack of studies examining the effects of consuming 20E as a dietary supplement in combination with HIIT on cardiovascular and pulmonary function outcomes in obese and overweight individuals. This research gap presents an opportunity to explore potential alternative strategies to reduce the risk of chronic cardiovascular diseases (e.g., hypertension and atherosclerosis) and chronic restrictive lung diseases in obese and overweight individuals. Therefore, this study examined the effects of HIIT combined with *A. officinalis* extract as a dietary supplement on cardiovascular and pulmonary function parameters as primary outcomes in obese and overweight individuals. In addition, body composition parameters were also examined as secondary outcomes.

## 2. Materials and Methods

### 2.1. Study Design

**Figure 1:**
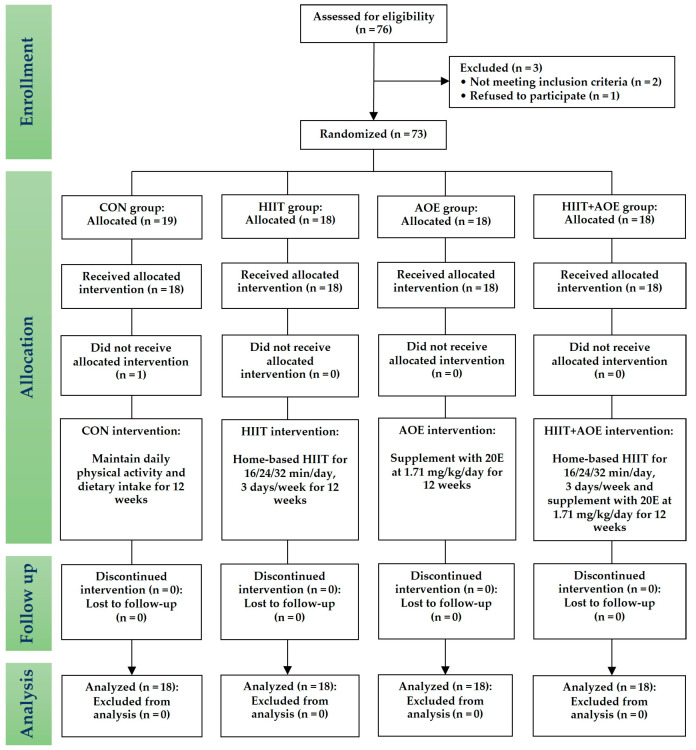
CONSORT flow diagram.

This randomized controlled trial started in September 2022 and recruited obese (body mass index (BMI) > 24.9 kg/m^2^) and overweight (BMI 23–24.9 kg/m^2^) individuals living in Chonburi Province [28]. As illustrated by the CONSORT flow diagram (Figure 1), a stratified blocked randomization method was used to assign eligible participants to one of four groups through the RAND function in Microsoft Excel version 2016 program: the control (CON) group, the HIIT group, the *A. officinalis* extract (AOE) group, or the HIIT combined with AOE (HIIT + AOE) group. This study was non-blinded, and the same researcher (T.P.) oversaw the screening, randomization, allocation, data collection, and analysis. To minimize potential selection, measurement, and information biases associated with data collection by a single researcher, stratified randomization and standardized objective variable collection were implemented. Additionally, a second researcher (P.P.) supervised all phases of the trial to ensure methodological rigor.

This study was registered in the Thai Clinical Trials Registry (ID: TCTR20220518001) and approved by the Burapha University Institutional Review Board on 24 May 2022 (ID: G-HS018/2565). Participants were provided with and signed an informed consent form before screening.

### 2.2. Screening of Participants

The inclusion criteria for the participants in this study were as follows: male or female, aged 18 to 30 years, and BMI > 22.9 kg/m^2^. Participants were further classified as overweight if their BMI ranged from 23.0 to 24.9 kg/m^2^, and as obese if their BMI was greater than 24.9 kg/m^2^ [28]. The exclusion criteria were daily supplementation with drugs or dietary supplements, food allergies—especially shoots or bulbs such as asparagus, bamboo shoots, green onions, onions, leeks, garlic bulbs, and chives—drug allergies, and the use of lithium drugs (e.g., lithium carbonate). Additional exclusion factors were pregnancy or breastfeeding, regular smoking (>30 packs/year), regular alcohol consumption (>1 cup/day), and a history of drug use or cardiovascular, liver, renal, musculoskeletal, infectious, cancer, neurological, or psychiatric disorders. Participants were withdrawn from the study if they experienced adverse symptoms during testing (e.g., nausea, vomiting, or fainting), reported serious study-related adverse effects (e.g., hospitalization due to exercise or supplementation), or chose to withdraw voluntarily.

### 2.3. Sample Size

The sample size was determined based on our pilot study involving eight individuals who were obese or overweight, divided into four groups of two participants each. The mean change in expiratory muscle strength (MEP) for each group was calculated as follows: CON, −3.60 cm H_2_O; HIIT, 7.60 cm H_2_O; AOE, 1.60 cm H_2_O; and HIIT + AOE, 7.40 cm H_2_O. The standard error of the mean across all groups was 3.00. The required sample size was calculated with the G*Power version 3.1.9.4 program using an alpha error of 0.05 and a test power of 0.95 [29]. Accounting for a 10% dropout rate, 18 participants per group were required, resulting in a total sample size of 72 individuals who were obese or overweight.

### 2.4. HIIT

This study utilized home-based bodyweight-bearing HIIT that was modified from a Tabata-style program, based on the protocol established in our previous study [22]. Each session comprised 4 min exercise cycles, alternating between 20 s of activity and 10 s of rest, followed by an additional 4 min active rest period, resulting in an 8 min cycle. Participants trained three times per week over 12 weeks, beginning with two cycles during the first four weeks, progressing to three cycles from weeks 5 to 8, and completing four cycles from weeks 9 to 12. Exercise intensity was maintained at 75–85% of maximum perceived exertion, with active rest periods involving arm swings at 40–50% exertion.

Participants in the HIIT and HIIT + AOE groups received an exercise diagram and video demonstration. The researcher monitored posture and compliance and provided real-time feedback. Sessions were scheduled individually, with communication via online platforms such as Line version 9.7.0 program, Microsoft Teams version 23257.2620.2442.7817 program, or Google Meet (https://meet.google.com, accessed in 1 May 2023). Exercise intensity was measured using Borg’s RPE scale (6–20) [30]. Participants were trained in its use before data collection and reported their exertion levels during home exercises. During each session, the exercise video played while the researcher corrected the participant’s posture in real time, recorded RPE scores, and provided real-time encouragement to maintain the target intensity.

### 2.5. Supplement

Preparation of the *A. officinalis* extract was conducted at the Faculty of Liberal Arts and Science, Kasetsart University, Nakhon Pathom, Thailand. In brief, hard stems of *A. officinalis* were collected from Nakhon Pathom, Thailand, from April to May 2022 and transported to the laboratory within one day. The samples were washed, rewashed in an ultrasonic bath for 10 min, chopped into 5 mm pieces, and oven-dried for 30 h at 60 °C to achieve a consistent weight with 5% moisture content. The dried *A. officinalis* was powdered, extracted with 95% ethanol for 3 days (repeated twice), filtered, and evaporated to dryness. The residue was suspended in water and analyzed using HPLC chromatography with a C18 Sep-Pak cartridge (Agilent Technologies, Waldbronn, Germany) at 40 °C with a 1 mL/min flow rate and a 20:80 acetonitrile/H_2_O mobile phase. The 20E compound was identified using UV absorbance at 245 nm, retention time, and spectral matching. A previously described method was used to quantify 20E [25,31]. During capsule preparation, capsules were filled with the *A. officinalis* extract powder under aseptic conditions, with each capsule weighing 500 mg. Each capsule contained 32.2 mg of 20E per gram of dry weight. The capsules were shipped frozen to Chonburi Province and stored at −20 °C in a freezer for subsequent experiments.

To monitor the intake of the supplement, the researchers invited participants in the AOE and HIIT + AOE groups to join designated Line application groups specific to their respective groups. Following pre-experiment data collection, participants received the capsules for home consumption. The researcher provided daily reminders via the Line group and instructed participants to record their supplement intake twice daily—once in the morning and once in the evening—using the group’s note function. Compliance with supplement intake was monitored and documented by the same researcher throughout the 12-week study period.

### 2.6. Experiments

The participants in each group were asked to perform the following during the 12 weeks of experiments:

1. Participants in the CON group maintained their daily physical activity and dietary intake.
2. Participants in the HIIT group performed a home-based HIIT program for 3 days/week.
3. Participants in the AOE group took *A. officinalis* extract powder capsules containing 20E at 1.71 ± 0.24 mg/kg/day daily after a meal (1 or 2 capsules for breakfast and 2 capsules for dinner) [24,32].
4. Participants in the HIIT + AOE group performed a home-based HIIT program for 3 days/week and took *A. officinalis* extract powder capsules containing 20E at 1.71 ± 0.24 mg/kg/day daily after a meal (1 or 2 capsules for breakfast and 2 capsules for dinner) [24,32].

### 2.7. Study End Points

**Figure 2:**
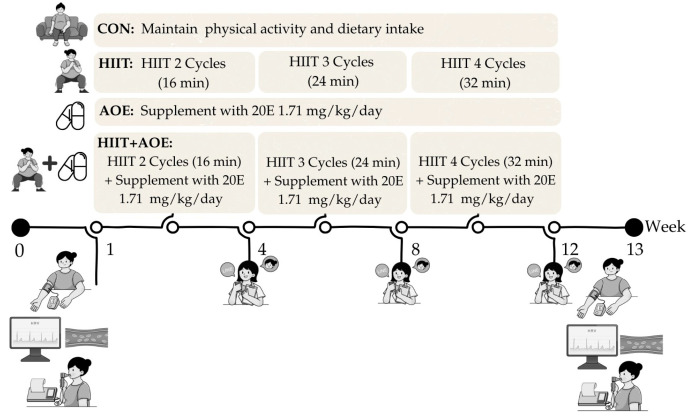
Experimental design and timeline of the 12-week intervention study. Participants were assigned to one of four groups: control (CON), HIIT (HIIT), *A. officinalis* extract (AOE), or HIIT combined with *A. officinalis* extract (HIIT+AOE). HIIT intensity progressed every 4 weeks, and AOE was orally administered daily at a dose of 1.71 mg/kg. Cardiopulmonary assessments were conducted before (Week 0) and after the intervention (Week 13), with interim check-ins at Weeks 4, 8, and 12.

The primary outcomes included cardiovascular function parameters, including HR, HR variability, endothelial function, BP, and BP variability, as well as pulmonary function parameters, including static and dynamic lung volume, respiratory muscle strength, and chest wall expansion. In addition, the secondary outcomes comprised body composition parameters in order to determine the cumulative impacts of the independent variables (i.e., HIIT program and *A. officinalis* extract supplementation). All outcomes were measured before and after the 12-week intervention by the same researcher, a certified physical therapist. Measurements were conducted between 08:00 and 12:00 on the appointment date at the Exercise and Nutrition Innovation and Sciences Research Unit Room, Faculty of Allied Health Sciences, Burapha University, Chonburi Province. The order of measurements over the study period is outlined in Figure 2.

#### 2.7.1. HR Variability

Measuring HR variability is a non-invasive method of assessing autonomic nervous system control over HR. In this study, short-term HR variability was applied and recorded for 10 min using Lead II electrocardiography (PowerLab^®^ 4/30, ADInstruments, New South Wales, Australia) and analyzed through both time-domain (the standard deviation of normal beat-to-beat (R-R) intervals (SDNN) and the root mean square of successive RR intervals (RMSSD)) and frequency-domain (total power (TP), very low-frequency (VLF) power, low-frequency (LF) power, high-frequency (HF) power, and LF/HF ratio) parameters [33] using the HR variability module (LabChart^®^ Pro, ADInstruments, New South Wales, Australia). These HR variability measures reflect the activities of the sympathetic and parasympathetic nervous systems and their balance, which control the heart [34].

#### 2.7.2. Endothelial Function

Endothelial function was assessed using the endothelium-dependent vasodilation technique [35]. Forearm blood flow was measured with the Laser Doppler Flowmetry Module (LDF100C, BIOPAC Systems Inc., Goleta, CA, USA) with the probe positioned perpendicularly to the brachial artery of the dominant arm. To measure blood flow during occlusion and recovery, a cuff from a standard mercury sphygmomanometer (Spirit™ CK-101, New Taipei City, Taiwan) was placed around the arm just above the probe. The cuff was inflated to approximately 200 mm Hg to fully occlude the brachial artery and then gradually deflated. Forearm blood flow was recorded during three phases—at rest, during occlusion, and after occlusion, each lasting 5 min—and is reported in perfusion units. The following parameters were analyzed: resting blood flow, blood flow during occlusion, peak blood flow after occlusion, the peak blood flow/resting blood flow ratio, and recovery time after occlusion. Recovery time is defined as the duration after occlusion when blood flow returns to levels similar to the resting value.

#### 2.7.3. BP and BP Variability

After a 10 min rest period, BP and HR were measured with the individual in the supine position using a digital automatic BP monitor (HEM-7121, Omron Healthcare Co., Ltd., Kyoto, Japan), which provided BP values with ± 3 mmHg accuracy and supported an arm cuff circumference of 22–32 cm. The BP cuff thoroughly enveloped the arm, and the inferior edge of the cuff was nearly 1 inch above the elbow crease. BP and HR were measured three times at 1 min intervals, and the mean of these three readings was recorded as the final result for systolic BP (SBP), diastolic BP (DBP), and HR values.

The pulse pressure (PP: SBP − DBP), mean arterial pressure (MAP: DBP + (PP/3)), and rate–pressure product (RPP: HR × SBP) were calculated based on the SBP, DBP, and HR values. Very short-term beat-to-beat BPV was assessed using the coefficient of variation for SBP and DBP (SBP CV and DBP CV, respectively). SBP CV was calculated as the standard deviation (SD) of the three SBP readings divided by the average SBP and multiplied by 100. Similarly, DBP CV was determined by dividing the SD of the three DBP readings by the mean DBP and multiplying by 100.

#### 2.7.4. Static and Dynamic Lung Volume and Capacity

Static lung volumes (including tidal volume (TV), vital capacity (VC), inspiratory capacity (IC), expiratory reserve volume (ERV), and inspiratory reserve volume (IRV)), as well as dynamic lung capacities (including forced expiratory volume in one second (FEV_1_), forced vital capacity (FVC), FEV_1_/FVC ratio, maximal voluntary ventilation (MVV), peak inspiratory and expiratory flow rate (PIF and PEF), and forced expiratory time (FET)), were measured using a portable automated spirometer (MicroLab, Micro Medical^®^, Kent, UK). Participants were instructed to perform two maneuvers: (1) maximum inspiration followed by slow and complete exhalation to assess slow vital capacity, and (2) maximum inspiration followed by rapid and complete exhalation to assess forced vital capacity. All measurements were conducted in accordance with the standardized guidelines of the American Thoracic Society and the European Respiratory Society [36].

#### 2.7.5. Respiratory Muscle Strength

Inspiratory and expiratory muscle strength were determined by measuring maximum inspiratory pressure (MIP) and maximum expiratory pressure (MEP) through the mouth, respectively, using a portable respiratory pressure meter (MicroRPM, CareFusion, Berkshire, UK). Participants were asked to perform maximum inspiration and rapidly and completely exhale for MEP, as well as perform maximum expiration and completely inhale for MIP. The measurement procedure followed the standard guidelines of the ATS/ERS Statement [37].

#### 2.7.6. Chest Wall Expansion

Expansion of the chest wall was circumferentially measured using a standard measuring tape in centimeters at the three following levels: the 3rd intercostal space (upper), the 5th intercostal space (middle), and the tip of the xyphoid process (lower) [38]. The difference between deep expiration and deep inspiration was measured twice. Three measurements were taken at each level, and the average of the three readings is reported. Participants were in the sitting position with their elbows slightly flexed so that their hands rested on their hips. Intra-tester reliability of upper-, middle-, and lower-chest expansion measurements was evaluated with five obese participants. Measurements of each participant were performed for 3 days. The intra-class correlation coefficient (ICC) shows good intra-tester reliabilities of upper- and lower-chest expansions (ICC = 0.88 and 0.83, respectively) and moderate intra-tester reliability for middle-chest expansion (ICC = 0.72) [39].

#### 2.7.7. Body Composition

Body composition parameters, including BM, BMI, skeletal muscle mass, fat-free mass, fat mass, percent body fat, waist circumference, waist–hip ratio, and basal metabolic rate, were assessed using a bioelectrical impedance analyzer (InBody270, InBody Co., Ltd., Daejeon, Republic of Korea).

#### 2.7.8. Physical Activity

To minimize the influence of external factors, all participants were instructed to maintain their routine physical activities and dietary intake throughout the study period. The Thai version of the Baecke Habitual Physical Activity Questionnaire was also used to assess participants’ activity levels, categorizing them as sedentary or physically active [40,41].

#### 2.7.9. Feasibility

The completion and dropout rates were recorded for all groups. Additionally, compliance (number of HIIT sessions completed), adherence (number of participants who reached the target exertion level during HIIT), and RPE were documented to assess exercise intensity during the HIIT program in the HIIT and HIIT + AOE groups. For the AOE and HIIT + AOE groups, the number of supplement capsules consumed over the 12-week study period was recorded.

### 2.8. Statistical Analysis

Data were analyzed using SPSS version 26 (IBM Corp., Armonk, NY, USA) and are presented as mean ± SD or range. The Kolmogorov–Smirnov test was used to assess the normality of data distributions, and appropriate parametric or non-parametric tests were applied accordingly. Between-group differences were analyzed using the one-way analysis of variance (ANOVA), followed by Bonferroni post hoc testing. Since body composition, vital signs, and pulmonary function data did not follow a normal distribution, they were analyzed using the Kruskal–Wallis test, followed by the Mann–Whitney U test for pairwise comparisons. Except for endothelial function values, some participants’ post-test data could not be recorded due to technical issues with the instrument. As a result, an intention-to-treat analysis was employed to account for missing data. Within-group differences between pre- and post-test measurements were assessed using the paired *t*-test. Statistical significance was set at *p* < 0.05. The effect size (*ES*) was calculated following Cohen’s guidelines, with values above 0.8 denoting large effects, 0.5–0.8 medium effects, and 0.2–0.5 small effects.

## 3. Results

### 3.1. Participant Characteristics and Feasibility

Seventy-six participants who were obese or overweight voluntarily participated in the study; however, only seventy-three met the inclusion criteria and were enrolled. Of these, seventy-two participants completed the study, as one participant from the control group declined participation. The final participants consisted of seventy-two participants (fifty-eight females, 80.60%) classified as obese (*n* = 55, 23.6%) or overweight (*n* = 17, 76.4%). The characteristics of the participants in each group are presented in Table 1. There were no statistically significant differences in baseline physical characteristics between the four groups.

The exercise dropout rate remained at zero percent in both the HIIT and HIIT + AOE groups, and no serious adverse events occurred during the trial. The rating of perceived exertion during the home-based HIIT program ranged from 7 to 11 (extremely light to light intensity) during the warm-up phase, 13 to 17 (hard to very hard intensity) during the exercise phase, and 9 to 11 (light intensity) during the cool-down phase. A few minor adverse events were reported in both groups. Specifically, two participants from the HIIT group and two from the HIIT + AOE group experienced leg muscle soreness and nausea during the fourth cycle of the HIIT program. However, none of the participants discontinued the trial.

All participants in both the AOE and HIIT + AOE groups consumed *A. officinalis* extract capsules without experiencing any adverse effects or complications. The AOE group consumed an average of 245.94 ± 2.94 capsules, corresponding to 79.99 ± 11.48% of the prescribed supplement dosage, while the HIIT + AOE group consumed an average of 256.94 ± 2.83 capsules, equivalent to 80.24 ± 2.83% of the prescribed dosage.

Furthermore, throughout the 12-week intervention period, no significant changes in daily physical activity were observed over time, and no notable differences were detected between the groups (Table 1).

### 3.2. Effects on HR and HR Variability

Before the 12-week intervention, no significant differences were observed in HR or HR variability among the four groups. After the 12-week intervention, a significant reduction in LF power was found in the HIIT group (−12.34 ± 14.12 nu, *p* = 0.002) and the HIIT + AOE group (−6.54 ± 10.21 nu, *p* = 0.015) compared to the baseline. Similarly, the LF/HF ratio significantly decreased in both the HIIT group (−0.52 ± 0.75 nu, *p* = 0.010) and the HIIT + AOE group (−0.33 ± 0.66 nu, *p* = 0.048). Between-group comparisons after the intervention revealed that the HIIT + AOE group had significantly higher HF power (*p* = 0.038, *ES* = 0.51) as well as a significantly lower LF/HF ratio (*p* = 0.038, *ES* = 0.45) compared to the control group (Table 2).

### 3.3. Effects on Endothelial Function

**Figure 3:**
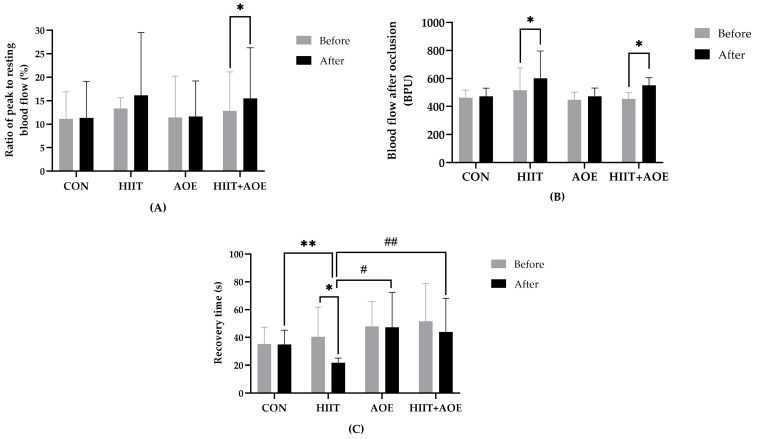
Ratio of peak blood flow after occlusion to resting blood flow (ratio of peak/baseline) (**A**), blood flow after occlusion (**B**), and recovery time after occlusion (**C**) of participants in control group (CON), HIIT group (HIIT), *A. officinalis* extract group (AOE), and HIIT combined with *A. officinalis* extract group (HIIT + AOE) before and after the 12-week intervention. *, *p* < 0.05 vs. before intervention; **, *p* < 0.05 vs. CON group; ^#^, *p* < 0.05 vs. AOE group; ^##^, *p* < 0.05 vs. HIIT + AOE group.

Before the intervention, no significant differences were observed in resting blood flow, blood flow during occlusion, peak blood flow after occlusion, the ratio of peak blood flow after occlusion to resting blood flow, or recovery time after occlusion. However, after the 12-week intervention, the HIIT group demonstrated a significant increase in peak blood flow after occlusion (86.09 ± 152.77 BPU, *p* = 0.003) (Figure 3B) and a shorter recovery time after occlusion (−18.66 ± 21.76 s, *p* = 0.004) (Figure 3C). Moreover, the HIIT + AOE group also exhibited a significant increase in peak blood flow after occlusion (96.23 ± 169.92 BPU, *p* = 0.028) (Figure 3B) and the ratio of peak blood flow after occlusion to resting blood flow (2.68 ± 7.06, *p* = 0.048) (Figure 3A).

When groups were compared, the HIIT group exhibited a significantly shorter recovery time after occlusion compared to the CON group (−13.19 ± 6.13 s, *p* = 0.02, *ES* = 0.65), AOE group (−25.27 ± 6.13 s, *p* < 0.001, *ES* = 0.57), and HIIT + AOE group (−22.19 ± 6.13 s, *p* < 0.01, *ES* = 0.54) (Figure 3C). No significant differences were observed in resting blood flow, blood flow during occlusion, peak blood flow after occlusion, the ratio of peak to resting blood flow, or recovery time between the CON and AOE groups or between the CON and HIIT + AOE groups.

### 3.4. Effects on BP and BP Variability

During the pre-test phase, no significant differences were observed in BP variables, namely, SBP, DBP, MAP, PP, and RPP. After 12 weeks, the HIIT group exhibited a significant reduction in SBP (−4.18 ± 8.80 mmHg, *p* = 0.03), DBP (−3.36 ± 7.94 mmHg, *p* = 0.045), MAP (−3.64 ± 7.68 mmHg, *p* = 0.030), and RPP (−772.96 ± 1814.83 mmHg/min, *p* = 0.044); however, no significant differences were observed compared to the CON group. No significant within-group changes were observed in either the AOE or HIIT + AOE group, nor were they significantly different from the CON group, as shown in Table 3.

**Figure 4:**
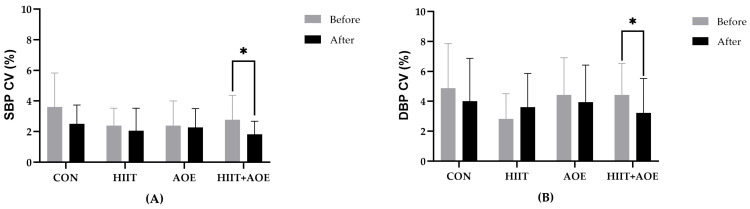
Very short-term blood pressure variability represented by the systolic blood pressure coefficient of variation (SBP CV) (**A**) and the diastolic blood pressure coefficient of variation (DBP CV) (**B**) of participants in control group (CON), HIIT group (HIIT), *A. officinalis* extract group (AOE), and HIIT combined with *A. officinalis* extract group (HIIT + AOE) before and after the 12-week intervention. *, *p* < 0.05 vs. before intervention.

For BPV variables, no significant differences were observed between the groups prior to the experiment. After 12 weeks, the HIIT + AOE group exhibited a reduction in SBP CV (−0.94 ± 1.73%, *p* = 0.033) and DBP CV (−1.2 ± 2.8%, *p* = 0.042); however, these changes were not significantly different from those in the CON group. Additionally, no significant changes were observed in the AOE and HIIT groups between the pre- and post-experiment phases, and no differences were found among groups after 12 weeks, as shown in Figure 4.

### 3.5. Effects on Pulmonary Function

Table 4 shows that participants in the HIIT + AOE group demonstrated improvements in pulmonary function, as indicated by significant increases of 3.44 ± 1.38% in the FEV_1_/FVC ratio (*p* = 0.047, *ES* = 0.12) and 3.94 ± 1.54% in the percent-predicted FEV_1_/FVC (*p* = 0.038, *ES* = 0.01) compared to the CON group.

Intra-group analysis also showed that the HIIT + AOE group had increases of 2.44 ± 1.69% in the FEV_1_/FVC ratio (*p* = 0.049), 2.83 ± 1.89% predicted in the percent-predicted FEV_1_/FVC ratio (*p* = 0.045), 0.78 ± 1.14 L/min in PIF (*p* = 0.01), and 0.49 ± 1.02 L/min (*p* = 0.018) and 7.72 ± 16.53 % predicted in PEF (*p* = 0.022). The AOE group had increases of 1.38 ± 2.76% in the FEV_1_/FVC ratio (*p* = 0.032), 1.00 ± 1.08 L/min in PIF (*p* = 0.001), 0.71 ± 1.10 L/min (*p* = 0.022), and 9.05 ± 18.31 % predicted in PEF (*p* = 0.049). Similarly, the HIIT group had increases of 1.27 ± 1.47 L/min in PIF (*p* = 0.002), 0.71 ± 0.82 L/min (*p* = 0.01), and 10.05 ± 11.68 % predicted in PEF (*p* = 0.002). In contrast, the CON group had decreases of 1.00 ± 2.02% in the FEV_1_/FVC ratio (*p* = 0.049) and 1.11 ± 2.08 % predicted in the percent-predicted FEV_1_/FVC ratio (*p* = 0.037).

### 3.6. Effects on Respiratory Muscle Strength

**Figure 5:**
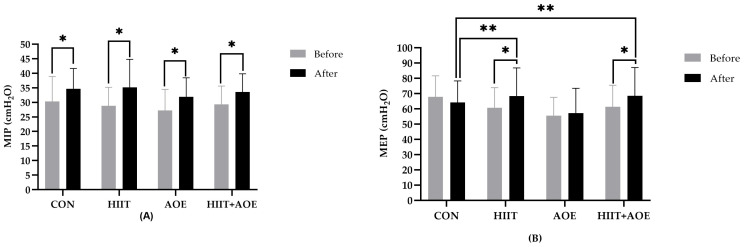
Maximal inspiratory pressure (MIP) (**A**) and maximal expiratory pressure (MEP) (**B**) of participants in control group (CON), HIIT group (HIIT), *A. officinalis* extract group (AOE), and HIIT combined with *A. officinalis* extract group (HIIT + AOE) before and after the 12-week intervention. *, *p* < 0.05 vs. before intervention; **, *p* < 0.05 vs. CON group.

Intra-group analysis showed that MIP improved in all four groups: the CON group (4.39 ± 3.17 cmH_2_O, *p* < 0.001), the HIIT group (6.31 ± 9.65 cmH_2_O, *p* = 0.03), the AOE group (4.71 ± 3.80 cmH_2_O, *p* <0.001), and the HIIT + AOE group (4.22 ± 3.27 cmH_2_O, *p* < 0.001) (Figure 5A). However, no differences were observed among groups.

Regarding expiratory muscle strength, MEP was improved only in the HIIT group (7.61 ± 13.71 cmH_2_O, *p* = 0.031) and the HIIT + AOE group (7.11 ± 13.05 cmH_2_O, *p* = 0.034). Moreover, it was significantly higher in the HIIT group (*p* = 0.029, *ES* = 0.12) and the HIIT + AOE group *(p* = 0.041, *ES* = 0.13) when compared to the CON group (Figure 5B).

### 3.7. Effects on Chest Wall Expansion and Pulmonary Volume

There were no significant differences in chest wall expansion among the four groups before the intervention. As shown in Table 5, the HIIT + AOE group had a significant improvement in lower-chest wall expansion, with an increase of 0.97 ± 1.26 cm (*p* = 0.005), which was 1.90 ± 0.48 cm greater than that in the CON group (*p* = 0.001, ES = 0.53). In addition, the HIIT group had an increase in upper-chest wall expansion of 0.82 ± 1.20 cm (*p* = 0.010). In contrast, neither the AOE group nor the CON group showed significant changes in chest wall expansion.

There were no significant differences in pulmonary volume indices between groups either before or after the 12-week intervention. However, the VC was improved in both the HIIT group (0.12 ± 0.20 L, *p* < 0.05) and the AOE group (0.18 ± 0.28 L, *p* < 0.05). Additionally, the AOE group also showed increases in IC (0.31 ± 0.33 L, *p* < 0.05) and IRV (0.27 ± 0.51 L, *p* < 0.05).

### 3.8. Effects on Body Composition

Table 6 presents the body composition parameters of participants in the CON, HIIT, AOE, and HIIT + AOE groups before and after the 12-week intervention. A significant increase in the waist–hip ratio was observed in both the CON group (Δ = 0.02 ± 0.04, *p* < 0.05) and the AOE group (Δ = 0.01 ± 0.03, *p* < 0.05) following the intervention. In contrast, the HIIT + AOE group demonstrated a significantly lower waist–hip ratio compared to the CON group after the intervention (*p* = 0.043, *ES* = 0.83). No other significant changes in body composition parameters were found in the groups.

## 4. Discussion

This study’s findings indicate that HR variability and endothelial function parameters were improved after a combination of HIIT and *A. officinalis* extract supplementation for 12 weeks. Furthermore, expiratory muscle strength was increased following this regimen, leading to increased forced exhalation, which better reflects lung emptying and enhanced lung expansion in participants who are obese or overweight. In addition, this intervention also helped maintain body proportions, such as the waist-to-hip ratio, as compared to the control intervention.

This study utilized doses of 20E that fell within both the established effective and safety ranges, with the actual participant doses adjusted for body weight and approximating 90–120 mg/day. The dosage selection was informed by existing literature, which reports that the median lethal dose of 20E in rodents exceeds 9 g/kg of body weight when administered orally, indicating a wide safety margin when transforming this dose for humans [32]. Additionally, previous studies involving individuals with metabolic syndrome, including those with obesity, have demonstrated that oral doses ranging from 50 to 200 mg/day are associated with beneficial physiological effects [24].

While 20E alone did not produce significant changes in the measured outcomes, it is known to influence molecular pathways related to protein synthesis, anti-inflammatory responses, and cardiovascular protection [24]. However, in the absence of a physiological stimulus such as exercise, these molecular effects may not manifest as measurable functional improvements. HIIT, on the other hand, activates multiple physiological systems, including metabolic cardiovascular and neuromuscular pathways [20,21], creating a responsive environment in which supplements like 20E can exert more pronounced effects. In this context, 20E may function to amplify or support the body’s adaptive responses, such as muscle protein remodeling, vascular function enhancement, and autonomic regulation, which are already initiated by HIIT.

### 4.1. Effects of HIIT Combined with A. officinalis Extract Supplementation on Cardiovascular Function Parameters

The results show that HIIT combined with *A. officinalis* extract (20E) supplementation improved endothelial function, as observed during hyperemic conditions. This may facilitate vascular adaptation to pressure fluctuations and thus reduce BP variability. Previous studies have not yet examined the effects of HIIT in combination with 20E supplementation. In a previous study examining the effects of a 4-week sprint/high-intensity interval training (sprint/HIIT) program—consisting of 4 to 7 constant-workload intervals at 200% of maximal power output, three times per week—in 16 obese men, the intervention was found to be effective in enhancing skeletal muscle capillarization, increasing endothelial nitric oxide synthase content, and reducing aortic stiffness [42]. According to a previous animal model study, HIIT comprising 14 repetitions of 20 s swimming sessions with 10 s rest intervals, four days per week for six weeks, significantly reduced central arterial stiffness—as assessed by arterial pulse-wave velocity—via an increase in aortic nitric oxide bioavailability [43]. These findings are consistent with our results, which were obtained by assessing arterial function using an endothelium-dependent vasodilation technique, and Laser Doppler measurements revealed an increase in peak blood flow following occlusion—indicating enhanced arterial vasodilation—in both the HIIT and HIIT + AOE interventions. Notably, the HIIT + AOE group exhibited a greater increase in peak blood flow, resulting in a higher ratio of peak post-occlusion blood flow to resting blood flow. On the other hand, the HIIT group demonstrated a faster vascular recovery time, suggesting predominantly improved vascular responsiveness.

Although studies regarding 20E in humans are still limited in some aspects, such as endothelial function, emerging evidence suggests its promising potential. Most investigations into the effects of 20E on endothelial function have been conducted in animal models such as mice, rats, and ovine [44]. For instance, a study on ovine found that 20E induced vasodilation in skeletal muscle arterioles via a nitric oxide-dependent mechanism that is independent of estrogen receptor β signaling. This suggests a vasorelaxant pathway for 20E [45]. Pharmacokinetic studies indicate that 20E is well tolerated in humans, and clinical investigations have shown that 20E has a good safety profile and may function as a multifunctional agent with the potential to modulate endothelial function, reduce vascular resistance, and enhance cardio-metabolic health [24]. To the best of our knowledge, our study is among the first to investigate the effects of 20E alone and in combination with HIIT on endothelial function. The findings indicate that 20E supplementation alone did not produce significant changes in endothelial function. However, when combined with HIIT, there was a markedly greater improvement. It is important to note that our study employed a relatively low dose of 1.71 mg/kg/day, which falls within the range considered safe for human use. Higher doses of 20E may elicit different effects, but potential toxicity at increased levels must be carefully considered.

In this study, resting BP decreased only in the HIIT intervention, with statistically significant reductions of −4.18 ± 8.80 mmHg in SBP and −3.36 ± 7.94 mmHg in DBP. These diminutions are near clinically significant thresholds, which are defined as a reduction of at least 5 mmHg in SBP or a reduction of at least 2 mmHg in DBP [46,47]. These findings are consistent with a previous study by Lu et al. (2023) [48], who investigated the effects of a 12-week Tabata-style bodyweight functional HIIT program in 60 university students. Participants exercised for 13 min per session at 90% of their age-predicted maximum HR. The intervention reduced resting SBP from an average of 121.37 mmHg to 116.56 mmHg (a decrease of approximately 4–5 mmHg) compared to the control group. In the same study, a subgroup analysis of 10 overweight participants revealed a decrease in resting SBP from 121 mmHg to 117 mmHg (approximately 4 mmHg), with no change in DBP (71.6 mmHg pre- and post-intervention). Remarkably, our findings demonstrate a significant reduction in both SBP and DBP, which may be attributed to the longer and progressively increasing exercise duration used—16, 24, and 32 min every four weeks—potentially allowing for more pronounced cardiovascular adaptations [48]. These results indicate that HIIT alone can produce a clinically meaningful reduction in BP, which is associated with a lower risk of stroke, coronary heart disease, heart failure, major cardiovascular events, cardiovascular mortality, and all-cause mortality.

Meanwhile, 20E has demonstrated beneficial effects on BP in animal studies, particularly in spontaneously hypertensive rats. Treatment with 20E has been shown to lower BP via a hypolipidemic effect and prevent the development of dilated cardiac hypertrophy in these models [49]. A recent study investigating the effects of consuming approximately 30 mg/day of 20E extracted from the hard stems of asparagus—similar to the source used in our study—in combination with resistance exercise over a 12-week period in 20 male athletes showed a reduction in plasma cortisol levels [25]. This stress hormone induces vasoconstriction and impairs vascular function; thus, its reduction may enhance the susceptibility of blood vessels to dilation, potentially contributing to improved vascular function [50]. Obesity is usually linked to higher BP, both SBP and DBP. This may cause greater variations in BP readings, which could indicate an unstable cardiovascular system. Notably, when 20E supplementation was combined with HIIT, the vascular benefits were further amplified, suggesting a synergistic effect that may help reduce BP variability.

Data on HR variability, particularly in the frequency domain, e.g., LF power, HF power, or their ratio, show significant improvements in the HIIT and HIIT + AOE groups. Similarly, a previous study examining the effects of whole-body HIIT—consisting of 10 min of exercises such as burpees, mountain climbers, jumping jacks, and squats—on HR variability in 21 insufficiently active adults. This study showed significant increases in time-domain HRV parameters, specifically SDNN and RMSSD, while no significant changes were observed in any frequency-domain parameters [51]. Likewise, a previous investigation of 12-week HIIT on autonomic function in 38 young males revealed significant increases in HR variability parameters, specifically in both LF and HF power, indicating improved autonomic regulation of the heart [52]. Notably, changes in HRV following a HIIT program may exhibit either positive or negative effects, depending on physical, physiological, and external factors, such as the baseline physical activity level (sedentary, active, or deconditioned), individual stress response, and intensity and duration of the exercise intervention.

Research regarding the effects of 20E on HR variability also remains limited, highlighting the need for further studies to confirm existing and novel findings and to establish the optimal dosage and duration of 20E supplementation for improving autonomic function [53]. However, the current study suggests that 20E supplementation may be beneficial to cardiovascular health when combined with HIIT—particularly in improving HR variability, endothelial function, and BP regulation—although our findings do not indicate the direct effects of this supplement alone on cardiovascular outcomes.

A potential hypothesis for the physiological adaptations observed with the combination of HIIT and 20E supplementation is based on their complementary mechanisms of action. HIIT is known to induce significant metabolic stress, a key stimulus for muscle hypertrophy and cardiovascular adaptation [54]. Concurrently, 20E has been shown to enhance protein synthesis in skeletal muscle and cardiac tissue and to exert protective effects against experimental atherosclerosis in mammals [24]. These mechanisms may help explain the positive effects on cardiovascular function observed in this study. Nevertheless, this remains a theoretical explanation, and further research is required to confirm the specific biological adaptations involved.

### 4.2. Effects of HIIT Combined with A. officinalis Extract Supplementation on Pulmonary Function Parameters

Participants in the HIIT + AOE group had increased expiratory muscle strength as measured by MEP, leading to improved lower-chest wall compliance and lung function parameters, including FEV_1_/FVC, FEV_1_/FEV %predicted, PEF, and %PEF. This allows more lung emptying in individuals with obesity and overweight, who tend to experience restrictive lung issues. Based on a review of the existing literature, this study is likely the first to demonstrate that a combination of HIIT and *A. officinalis* extract supplementation significantly enhances respiratory muscle strength and pulmonary function. According to a previous study on HIIT, 12 weeks of HIIT (functional bodyweight Tabata/HIIT and cycling/HIIT) decreased waist-to-hip ratio by −0.02 cm and waist circumference by −3.7 cm in obesity and overweight [22,55], which, in turn, might expand lung volume and capacity within the abdominal cavity. This expansion also increases the space between chest ribs, resulting in an extension of the initial length of the expiratory muscles and thus improving the length–tension relationship of the muscle fibers [56].

This study is also the first to investigate the effects of 20E supplementation with HIIT on pulmonary function. Our findings indicate that the consumption of approximately 90 mg/day, or 1.71 mg/kg/day, of 20E with HIIT resulted in enhanced pulmonary function, as indicated by improvements in the FEV_1_/FVC ratio and percent-predicted FEV_1_/FVC. Currently, research on the effects of 20E on pulmonary function remains in the development phase in terms of therapeutic applications in humans, particularly among patients with pulmonary arterial hypertension and severe pneumonia. Preliminary evidence suggests that 20E may activate non-peptide receptors and exert anti-inflammatory, anti-thrombotic, and anti-fibrotic effects. These mechanisms are anticipated to improve pulmonary function and potentially enhance survival rates in affected patients [57]. The observed improvements in our study suggest a possible enhancement in airway ventilation, as indicated by increased FEV_1_/FEV% and PEF. This enhancement may be attributed to increased respiratory muscle strength, as evidenced by elevated MEP, as well as a reduction in airway constriction. These changes may be associated with reduced systemic inflammation and fibrosis linked to restrictive lung dysfunction in obesity, as indicated by the improved FEV_1_/FVC ratio. Although the effects of 20E on pulmonary health are still unclear and under investigation, existing findings are promising. Importantly, further research is warranted to elucidate the underlying mechanisms involved.

### 4.3. Effects of HIIT Combined with A. officinalis Extract Supplementation on Body Composition Parameters

Despite the growing popularity of HIIT for bodyweight and fat reduction, its effectiveness remains debatable. In the present study, a 12-week HIIT intervention did not significantly reduce BM, BMI, skeletal muscle mass, fat-free mass, fat mass, percent body fat, or the waist-to-hip ratio. These findings suggest that HIIT alone, with regard to this study’s regimen, may be insufficient to induce meaningful changes in body composition outcomes. This finding aligns with a previous study in which a 6-week Wingate sprint/HIIT program conducted with ten participants did not alter BM [58]. While many HIIT studies involving athletes and healthy adolescents or adults have demonstrated beneficial effects on BM and fat loss, evidence in populations with non-communicable diseases remains limited [19]. This may be due to the demanding nature of high-intensity protocols, which could pose challenges or risks to individuals with compromised health status.

Supplementation with 20E in overweight individuals has shown potential benefits in reducing BM and improving body composition indices. However, studies specifically investigating 20E derived from *A. officinalis* hard stems remain limited and yield inconclusive results. One previous study administering 20E at a dose of 100–200 mg/day (2 × 50 mg) over a three-month period reported 1.3%, 3.2%, and 7.6% reductions in BM, waist circumference, and body fat, respectively [26]. These outcomes differ from our findings, which may be attributable to the low dosage applied (approximately 90–120 mg of 20E/day).

Interestingly, when 20E was combined with HIIT, the waist-to-hip ratio was maintained, despite no significant changes in BM, fat mass, or muscle mass. This suggests a stabilization of body proportions, in contrast to the control group, which demonstrated an approximate 2% increase in the waist-to-hip ratio. Recent studies have highlighted the waist-to-hip ratio and waist circumference as more reliable predictors of cardiopulmonary health risks compared to the traditional measure, BMI. An elevated waist-to-hip ratio has been significantly associated with an increased risk of myocardial infarction (odds ratio of 1.98) [59,60]. Beyond cardiovascular implications, a higher waist-to-hip ratio is inversely associated with lung function metrics such as FVC and FEV_1_, indicating compromised pulmonary health [61]. These findings emphasize the importance of the waist-to-hip ratio as a critical anthropometric measure in assessing both cardiovascular and pulmonary health risks.

### 4.4. Limitations

There are some limitations in this study that should be addressed. Firstly, BP variability was assessed using a simple method with very short-term beat-to-beat measurements taken 10 min after the participants assumed a supine position, with three readings recorded at 1 min intervals. In clinical practice, Ambulatory Blood Pressure Monitoring (ABPM) conducted at 15 or 30 min intervals and Home Blood Pressure Monitoring (HBPM) are considered the gold-standard methods for assessing mid-term BP variability, offering high reliability for capturing day-to-day or week-to-week fluctuations. Therefore, it is recommended that future studies incorporate ABPM or HBPM to provide a more comprehensive assessment of BP variability. Secondly, participants were instructed to maintain their usual physical activity and dietary habits throughout the experimental period; however, complete control over their activity levels and dietary intake was not feasible, as they were ecologically valid. Nevertheless, baseline data indicated no significant differences in overall physical activity and dietary intake before and after the interventions or between groups. However, to minimize potential confounding factors, such as natural physiological fluctuations, learning effects, or other external factors (e.g., emotional arousal), future studies should incorporate a familiarization session prior to the actual measurements. Thirdly, this study did not examine molecular and biochemical factors that may influence BP and pulmonary function, such as blood lipid profiles, blood glucose levels, inflammatory mediators, or oxidative stress biomarkers. Therefore, further investigation of these parameters is recommended to provide a more comprehensive understanding of the underlying mechanisms. Lastly, no statistically significant change in vascular recovery time was observed in the HIIT + AOE group, which was observed only in the HIIT group. This result might have been influenced by individual intrinsic factors, particularly the variability in the collateral circulation of the upper arm vessels following occlusion. Differences in cuff size, placement, and vascular anatomy among the participants may have affected the results. Notably, this study did not assess the blood flow in vessels adjacent to the brachial artery, which represents a limitation. Future research should include measurements of collateral and surrounding vascular responses to provide a more comprehensive understanding.

## 5. Conclusions

This study indicated that a 12-week HIIT program combined with *A. officinalis* extract supplementation improved HR variability indices, potentially lowering cardiovascular disease and atherosclerosis risk via increased endothelial function. It also enhanced expiratory muscle strength, lower-chest wall expansion, and, consequently, pulmonary function. The clinical implication is that utilizing HIIT in conjunction with supplementation with *A. officinalis* extract containing 20E at 1.71 ± 0.24 mg/kg/day daily for 12 weeks is safe and could be an option for individuals who are obese or overweight. This combination has the potential to improve cardiovascular and respiratory function and serve as a preventive strategy against cardiovascular and respiratory disorders in obese and overweight individuals.

## Figures and Tables

**Table 1:** Baseline physical characteristics of participants in control group (CON), HIIT group (HIIT), *A. officinalis* extract group (AOE) (c), and HIIT combined with *A. officinalis* extract group (HIIT + AOE) before 12-week intervention.

	CON Group	HIIT Group	AOE Group	HIIT + AOE Group
Number	18	18	18	18
Sex (*n*, male–female)	5:13	5:13	2:16	2:16
Age (years)	21.61 ± 2.06	20.72 ± 1.32	20.22 ± 1.93	20.06 ± 2.01
WHO BMI classification (*n*, obese–overweight)	16:2	13:5	13:5	13:5
Physical activity level				
Sedentary (*n*, %)	3 (17%)	3 (17%)	3 (17%)	1 (6%)
Active (*n*, %)	11 (61%)	12 (66%)	9 (55%)	14 (78%)
Athletic (*n*, %)	4 (22%)	3 (17%)	6 (33%)	3 (17%)
Physical activity score				
Before	7.01 ± 1.07	7.04 ± 0.99	7.46 ± 1.19	6.86 ± 0.85
After	6.96 ± 0.79	7.31 ± 1.22	7.27 ± 0.99	7.37 ± 1.20

Data are presented as the number, percentage, ratio, and mean ± SD. BMI, body mass index; WHO, World Health Organization.

**Table 2:** Heart rate and its variability among participants in the control group (CON), HIIT group (HIIT), *A. officinalis* extract group (AOE), and HIIT combined with *A. officinalis* extract group (HIIT + AOE) before and after the 12-week intervention.

	CON Group (*n* = 18)		HIIT Group (*n* = 18)		AOE Group (*n* = 18)		HIIT + AOE Group (*n* = 18)	
	Before	After	Before	After	Before	After	Before	After
HR (beats/min)	76.53 ± 10.56	73.55 ± 11.04	77.95 ± 10.82	76.04 ± 10.03	75.03 ± 15.69	78.07 ± 9.94	74.09 ± 9.98	72.00 ± 8.25
NN interval (ms)	795.97 ± 93.24	831.07 ± 110.27	783.90 ± 110.24	803.33 ± 116.17	830.61 ± 161.17	781.56 ± 110.01	823.12 ± 106.74	844.26 ± 102.25
SDNN (ms)	56.42 ± 23.76	57.83 ± 21.09	48.79 ± 25.81	52.67 ± 20.47	62.02 ± 35.66	66.09 ± 45.50	62.77 ± 26.12	64.09 ± 26.12
RMSSD (ms)	48.36 ± 32.78	48.86 ± 22.86	39.32 ± 29.86	42.11 ± 25.69	64.15 ± 57.77	60.56 ± 65.05	56.22 ± 34.57	57.99 ± 35.70
Total power (ms^2^)	3536.15 ± 2982.59	3274.70 ± 2221.98	2965.62 ± 3132.98	2987.58 ± 2328.17	4895.11 ± 3803.14	6562.20 ± 4461.31	4397.41 ± 3847.59	4616.22 ± 4457.35
VLF power (ms^2^)	1377.50 ± 895.81	1564.83 ± 1024.58	1139.12 ± 1542.15	1182.67 ± 899.51	1366.86 ± 1100.79	2437.27 ± 2623.72	1678.00 ± 1474.66	1929.49 ± 1743.83
LF power (ms^2^)	799.42 ± 842.98	924.37 ± 448.48	715.01 ± 542.55	652.06 ± 462.84	953.64 ± 839.18	922.38 ± 3586.96	1170.22 ± 1492.73	963.91 ± 973.55
LF power (nu)	44.82 ± 18.09	49.09 ± 19.98	53.14 ± 13.72	40.80 ± 16.33 *	44.04 ± 21.70	40.23 ± 23.03	40.53 ± 16.38	33.99 ± 11.74 *
HF power (ms^2^)	1044.01 ± 1429.90	609.39 ± 478.49	1035.46 ± 1690.28	1168.29 ± 1165.66	2293.36 ± 3511.80	2467.31 ± 4393.79	1226.39 ± 985.26	1396.06 ± 1532.63
HF power (nu)	40.89 ± 17.65	39.06 ± 15.97	43.59 ± 17.03	49.54 ± 19.45	48.00 ± 21.62	50.75 ± 21.47	50.18 ± 15.37	55.47 ± 11.10 **
LF/HF ratio	1.65 ± 1.90	1.66 ± 1.31	1.58 ± 1.14	1.06 ± 0.81 *	1.63 ± 2.11	1.30 ± 1.39	1.00 ± 0.78	0.67 ± 0.36 *^,^**

Group comparisons were conducted using one-way ANOVA, followed by Bonferroni correction. Intra-group comparisons were performed using the Wilcoxon Signed-Rank test. HR, heart rate; NN, normal-to-normal beat; SDNN, standard deviation of normal beat-to-normal beat interval; RMSSD, root mean square of successive R-R interval difference; VLF, very low frequency; LF, low frequency; HF, high frequency; nu, normalized units. *, *p* < 0.05 vs. before intervention; **, *p* < 0.05 vs. CON group.

**Table 3:** Blood pressure variables of participants in control group (CON), HIIT group (HIIT), *A. officinalis* extract group (AOE), and HIIT combined with *A. officinalis* extract group (HIIT + AOE) before and after the 12-week intervention.

	CON Group (*n* = 18)		HIIT Group (*n* = 18)		AOE Group (*n* = 18)		HIIT + AOE Group (*n* = 18)	
	Before	After	Before	After	Before	After	Before	After
SBP (mmHg)	118.64 ± 16.15	112.42 ± 12.28	118.31 ± 11.77	114.12 ± 12.27 *	112.33 ± 13.24	112.46 ± 12.56	110.70 ± 8.08	111.01 ± 9.17
DBP (mmHg)	74.40 ± 12.53	70.64 ± 8,14	73.94 ± 8.54	70.57 ± 8.72 *	70.81 ± 11.46	71.72 ± 10.35	69.77 ± 7.16	69.24 ± 6.58
MAP (mmHg)	89.15 ± 13.03	84.57 ± 8.74	88.73 ± 8.99	85.09 ± 9.05 *	84.65 ± 11.34	85.30 ± 10.57	83.42 ± 7.16	83.16 ± 7.10
PP (mmHg)	44.24 ± 9.91	41.77 ± 9.00	44.37 ± 7.89	43.55 ± 9.25	41.51 ± 8.85	40.74 ± 7.44	40.92 ± 4.61	41.77 ± 5.41
RPP (mmHg/min)	9156.46 ± 1604.82	8170.73 ± 1372.36	9147.75 ± 1704.77	8374.79 ± 1358.85 *	8433.38 ± 2621.42	8043.40 ± 1408.16	8647.23 ± 1093.37	8179.88 ± 1589.57

Group comparisons were conducted using one-way ANOVA, followed by Bonferroni correction. Intra-group comparisons were performed using paired *t*-test. SBP, systolic blood pressure; DBP, diastolic blood pressure; MAP, mean arterial pressure; PP, pulse pressure; RPP, rate–pressure product. *, *p* < 0.05 vs. before intervention.

**Table 4:** Pulmonary function of participants in control group (CON), HIIT group (HIIT), *A. officinalis* extract group (AOE), and HIIT combined with *A. officinalis* extract group (HIIT + AOE) before and after the 12-week intervention.

	CON Group (*n* = 18)		HIIT Group (*n* = 18)		AOE Group (*n* = 18)		HIIT + AOE Group (*n* = 18)	
	Before	After	Before	After	Before	After	Before	After
Forced expiratory volume in one second (FEV_1_)								
FEV_1_ (L)	2.89 ± 0.52	2.87 ± 0.50	3.02 ± 0.87	3.06 ± 0.85	2.92 ± 0.59	3.00 ± 0.52	2.88 ± 0.54	2.94 ± 0.50
FEV_1_ (%predicted)	95.72 ± 9.81	95.44 ± 10.01	98.88 ± 11.58	100.44 ± 11.50	99.61 ± 15.95	101.11 ± 13.23	99.16 ± 14.27	101.44 ± 10.52
Forced vital capacity (FVC)								
FVC (L)	3.19 ± 0.65	3.21 ± 0.63	3.39 ± 1.08	3.44 ± 1.04	3.28 ± 0.64	3.34 ± 0.58	3.26 ± 0.58	3.26 ± 0.66
FVC (%predicted)	93.55 ± 8.16	94.22 ± 9.34	98.50 ± 13.09	100.11 ± 12.41	99.83 ± 14.68	100.00 ± 13.55	100.77 ± 11.55	100.67 ± 12.46
Peak expiratory flow (PEF)								
PEF (L/min)	6.95 ± 1.64	6.98 ± 1.62	6.49 ± 1.39	7.20 ± 1.41 *	6.44 ± 1.79	7.15 ± 1.53 *	6.24 ± 1.18	6.74 ± 1.12 *
PEF (%predicted)	97.27 ± 25.16	97.50 ± 23.90	91.50 ± 15.45	101.56 ± 15.06 *	92.27 ± 19.14	101.33 ± 16.78 *	92.11 ± 12.87	99.83 ± 14.72 *
Forced expiratory volume in one second per forced vital capacity (FEV_1_/FVC)								
FEV_1_/FVC (%)	90.77 ± 5.63	89.77 ± 5.57 *	89.72 ± 5.54	89.61 ± 4.85	89.38 ± 6.89	90.33 ± 6.53 *	88.72 ± 9.28	91.17 ± 5.34 *^,^**
FEV_1_/FVC (%predicted)	101.05 ± 5.57	99.94 ± 5.59 *	99.55 ± 5.92	99.50 ± 5.03	98.83 ± 7.48	100.22 ± 7.22	97.77 ± 10.14	100.61 ± 6.00 *^,^**
Forced expiratory flow at 25–75% of FVC (FEF_25-75_)								
FEF_25-75_ (L)	3.71 ± 0.96	3.58 ± 0.89	3.67 ± 0.98	3.56 ± 0.76	3.61 ± 1.03	3.76 ± 0.95	3.46 ± 0.99	3.73 ± 0.60
FEF_25-75_ (%predicted)	98.00 ± 25.95	94.61 ± 24.24	96.00 ± 18.58	95.22 ± 17.82	97.1 ± 26.30	100.56 ± 24.68	94.11 ± 27.456	101.94 ± 18.35
Maximal voluntary ventilation (MVV) (L/min)	108.43 ± 19.59	107.85 ± 18.89	113.46 ± 32.73	114.92 ± 31.86	109.84 ± 22.43	112.88 ± 110.60	108.14 ± 20.25	110.60 ± 18.80
Peak inspiratory flow (PIF) (L/min)	4.71 ± 1.36	5.03 ± 1.31	4.22 ± 1.50	5.49 ± 1.61 *	4.17 ± 1.41	5.17 ± 1.41 *	4.11 ± 0.70	4.89 ± 1.21 *
Forced expiratory time (s)	2.40 ± 0.61	2.51 ± 0.65	2.72 ± 0.65	2.82 ± 0.64	2.58 ± 0.73	2.50 ± 0.79	2.88 ± 0.90	2.61 ± 0.85

Group comparisons were conducted using one-way ANOVA, followed by Bonferroni correction. Intra-group comparisons were performed using paired *t*-test. *, *p* < 0.05 vs. before intervention; **, *p* < 0.05 vs. CON group.

**Table 5:** Chest wall expansion and pulmonary volume of participants in control group (CON), HIIT group (HIIT), *A. officinalis* extract group (AOE), and HIIT combined with *A. officinalis* extract group (HIIT + AOE) before and after the 12-week intervention.

	CON Group (*n* = 18)		HIIT Group (*n* = 18)		AOE Group (*n* = 18)		HIIT + AOE Group (*n* = 18)	
	Before	After	Before	After	Before	After	Before	After
**Chest expansion**								
Upper chest (cm)	2.94 ± 1.49	3.02 ± 1.26	2.71 ± 1.11	3.54 ± 1.50 *	2.83 ± 0.79	3.30 ± 0.84	3.40 ± 0.83	3.62 ± 1.39
Middle chest (cm)	2.07 ± 1.45	2.21 ± 1.46	2.36 ± 1.36	2.88 ± 2.00	2.37 ± 1.02	2.98 ± 1.36	2.97 ± 1.10	3.22 ± 1.36
Lower chest (cm)	2.89 ± 1.52	2.88 ± 1.56	3.41 ± 1.30	3.85 ± 1.75	3.85 ± 1.46	3.71 ± 0.92	3.81 ± 0.97	4.79 ± 1.49 *^,^**
**Pulmonary volume**								
VC (L)	3.01 ± 0.73	3.10 ± 0.71	3.22 ± 1.16	3.34 ± 1.13 *	3.02 ± 0.62	3.21 ± 0.58 *	3.13 ± 0.69	3.17 ± 0.67
IC (L)	1.93 ± 0.05	2.00 ± 0.59	2.02 ± 0.64	2.17 ± 0.63	1.77 ± 0.64	2.08 ± 0.60 *	1.91 ± 0.30	1.96 ± 0.44
TV (L)	0.96 ± 0.38	0.86 ± 0.54	0.94 ± 0.47	0.99 ± 0.39	0.84 ± 0.35	0.88 ± 0.43	0.99 ± 0.41	0.95 ± 0.47
ERV (L)	1.12 ± 0.56	1.11 ± 0.51	1.19 ± 0.62	1.17 ± 0.58	1.25 ± 0.41	1.13 ± 0.38	1.22 ± 0.50	1.21 ± 0.42
IRV (L)	0.96 ± 0.47	1.15 ± 0.59	1.07 ± 0.52	1.19 ± 0.52	0.93 ± 0.60	1.20 ± 0.41 *	0.92 ± 0.31	1.01 ± 0.51

Group comparisons were conducted using one-way ANOVA, followed by Bonferroni correction. Intra-group comparisons were performed using paired *t*-test. VC, vital capacity; IC, inspiratory capacity; TV, tidal volume; ERV, expiratory reserve volume; IRV, inspiratory reserve volume. *, *p* < 0.05 vs. before intervention; **, *p* < 0.05 vs. CON group.

**Table 6:** Body composition of participants in control group (CON), HIIT group (HIIT), *A. officinalis* extract group (AOE) (c), and HIIT combined with *A. officinalis* extract group (HIIT + AOE) before and after the 12-week intervention.

	CON Group (*n* = 18)		HIIT Group (*n* = 18)		AOE Group (*n* = 18)		HIIT + AOE Group (*n* = 18)	
	Before	After	Before	After	Before	After	Before	After
Body mass (kg)	77.40 ± 15.25	77.36 ± 14.49	77.64 ± 16.60	78.89 ± 16.75	71.43 ± 13.05	72.71 ± 13.54	70.47 ± 7.60	71.19 ± 7.67
Body mass index (kg/m^2^)	29.10 ± 5.11	29.11 ± 4.87	29.13 ± 5.48	29.59 ± 5.65	26.98 ± 2.97	27.44 ± 3.04	26.92 ± 2.50	27.19 ± 2.32
Skeletal muscle mass (kg)	25.37 ± 5.52	25.25 ± 5.55	25.88 ± 6.08	26.14 ± 6.27	24.60 ± 5.79	24.79 ± 6.09	24.14 ± 3.97	23.96 ± 3.84
Fat-free mass (kg)	46.04 ± 9.06	45.82 ± 9.06	46.85 ± 9.84	47.24 ± 10.20	44.73 ± 9.60	45.11 ± 10.12	44.12 ± 6.50	43.77 ± 6.28
Fat mass (kg)	31.36 ± 10.23	31.53 ± 9.62	30.79 ± 11.64	31.65 ± 11.85	26.70 ± 6.21	27.60 ± 6.52	26.34 ± 5.26	27.42 ± 4.84
Percent body fat (%)	40.07 ± 7.38	40.44 ± 7.14	39.02 ± 8.98	39.54 ± 9.21	37.44 ± 5.53	38.04 ± 5.64	37.37 ± 5.79	38.52 ± 5.10
Waist circumference (cm)	95.13 ± 9.53	95.41 ± 11.75	95.31 ± 12.97	94.38 ± 12.99	87.73 ± 9.92	89.28 ± 9.44	85.04 ± 8.13	86.96 ± 6.99
Waist–hip ratio	0.92 ± 0.06	0.94 ± 0.06 *	0.91 ± 0.06	0.91 ± 0.06	0.91 ± 0.15	0.93 ± 0.81	0.87 ± 0.04	0.88 ± 0.05 **
Basal metabolic rate (kcal)	1364 ± 195	1359 ± 196	1382 ± 212	1390 ± 220	1336 ± 207	1344 ± 218	1323 ± 140	1315 ± 135

Group comparisons were conducted using one-way ANOVA, followed by Bonferroni correction. Intra-group comparisons were performed using paired *t*-test. *, *p* < 0.05 vs. before intervention; **, *p* < 0.05 vs. CON group.

## Data Availability

The data are available upon request from the corresponding author.

## Abbreviations

The following abbreviations are used in this manuscript:

20E	20-Hydroxyecdysone
AOE	*A. officinalis* extract group
BMI	Body mass index
BP	Blood pressure
BPV	Blood pressure variability
COM	Combined group
CON	Control group
CV	Coefficient of variation
DBP	Diastolic blood pressure
ERV	Expiratory reserve volume
ES	Effect size
FET	Forced expiratory time
FEV_1_	Forced expiratory volume in one second
FRC	Functional residual capacity
FVC	Forced vital capacity
HF	High frequency
HIIT	High-intensity intermittent training
HPLC	High-performance liquid chromatography
HR	Heart rate
HRV	Heart rate variability
ICC	Intra-class correlation coefficient
IRV	Inspiratory reserve volume
LF	Low frequency
MAP	Mean arterial pressure
MEP	Maximum expiratory mouth pressure
MIP	Maximum inspiratory mouth pressure
MVV	Maximal voluntary ventilation
PEF	Peak expiratory flow rate
PIF	Peak inspiratory flow rate
PP	Pulse pressure
RMSSD	Root mean square of successive RR interval
RPE	Rating of perceived exertion
RPP	Rate–pressure product
SD	Standard deviation
SDNN	Standard deviation of normal beat-to-beat
TP	Total power
TV	Tidal volume
UV	Ultraviolet
VC	Vital capacity
VLF	Very low frequency

## References

[1] Collaborators G.F. (2024). Burden of disease scenarios for 204 countries and territories, 2022–2050: A forecasting analysis for the Global Burden of Disease Study 2021. Lancet.

[2] Ansari S., Haboubi H., Haboubi N. (2020). Adult obesity complications: Challenges and clinical impact. Ther. Adv. Endocrinol. Metab..

[3] Kaufman C.L., Kaiser D.R., Steinberger J., Kelly A.S., Dengel D.R. (2007). Relationships of cardiac autonomic function with metabolic abnormalities in childhood obesity. Obesity.

[4] Tadic M., Cuspidi C., Vukomanovic V., Kocijancic V., Celic V., Stanisavljevic D. (2016). The Association between Obesity, Blood Pressure Variability, and Right Ventricular Function and Mechanics in Hypertensive Patients. J. Am. Soc. Echocardiogr..

[5] Yadav R.L., Yadav P.K., Yadav L.K., Agrawal K., Sah S.K., Islam M.N. (2017). Association between obesity and heart rate variability indices: An intuition toward cardiac autonomic alteration–a risk of CVD. Diabetes Metab. Syndr. Obes..

[6] Pelosi P., Croci M., Ravagnan I., Tredici S., Pedoto A., Lissoni A., Gattinoni L. (1998). The effects of body mass on lung volumes, respiratory mechanics, and gas exchange during general anesthesia. Anesth. Analg..

[7] Hedenstierna G., Santesson J. (1976). Breathing mechanics, dead space and gas exchange in the extremely obese, breathing spontaneously and during anaesthesia with intermittent positive pressure ventilation. Acta Anaesthesiol. Scand..

[8] Sharp J.T., Henry J.P., Sweany S.K., Meadows W.R., Pietras R.J. (1964). The total work of breathing in normal and obese men. J. Clin. Investig..

[9] Naimark A., Cherniack R.M. (1960). Compliance of the respiratory system and its components in health and obesity. J. Appl. Physiol..

[10] Schachter L.M., Salome C.M., Peat J.K., Woolcock A.J. (2001). Obesity is a risk for asthma and wheeze but not airway hyperresponsiveness. Thorax.

[11] Jones R.L., Nzekwu M.M. (2006). The effects of body mass index on lung volumes. Chest.

[12] Sin D.D., Jones R.L., Man S.F. (2002). Obesity is a risk factor for dyspnea but not for airflow obstruction. Arch. Intern. Med..

[13] Zerah F., Harf A., Perlemuter L., Lorino H., Lorino A.M., Atlan G. (1993). Effects of obesity on respiratory resistance. Chest.

[14] Lazarus R., Sparrow D., Weiss S.T. (1997). Effects of obesity and fat distribution on ventilatory function: The normative aging study. Chest.

[15] Biring M.S., Lewis M.I., Liu J.T., Mohsenifar Z. (1999). Pulmonary physiologic changes of morbid obesity. Am. J. Med. Sci..

[16] Bull F.C., Al-Ansari S.S., Biddle S., Borodulin K., Buman M.P., Cardon G., Carty C., Chaput J.P., Chastin S., Chou R. (2020). World Health Organization 2020 guidelines on physical activity and sedentary behaviour. Br. J. Sports Med..

[17] American College of Sports M., Liguori G., Feito Y., Fountaine C., Roy B.A. (2022). ACSM’s Guidelines for Exercise Testing and Prescription.

[18] Tschakert G., Hofmann P. (2013). High-Intensity Intermittent Exercise: Methodological and Physiological Aspects. Int. J. Sports Physiol. Perform..

[19] Boutcher S.H. (2011). High-intensity intermittent exercise and fat loss. J. Obes..

[20] Tabata I., Irisawa K., Kouzaki M., Nishimura K., Ogita F., Miyachi M. (1997). Metabolic profile of high intensity intermittent exercises. Med. Sci. Sports Exerc..

[21] Tabata I. (2019). Tabata training: One of the most energetically effective high-intensity intermittent training methods. J. Physiol. Sci..

[22] Padkao T., Prasertsri P. (2025). The Impact of Modified Tabata Training on Segmental Fat Accumulation, Muscle Mass, Muscle Thickness, and Physical and Cardiorespiratory Fitness in Overweight and Obese Participants: A Randomized Control Trial. Sports.

[23] Lafont R., Balducci C., Dinan L. (2021). Ecdysteroids. Encyclopedia.

[24] Dinan L., Dioh W., Veillet S., Lafont R. (2021). 20-Hydroxyecdysone, from Plant Extracts to Clinical Use: Therapeutic Potential for the Treatment of Neuromuscular, Cardio-Metabolic and Respiratory Diseases. Biomedicines.

[25] Denben B., Sripinyowanich S., Ruangthai R., Phoemsapthawee J. (2023). Beneficial Effects of Asparagus officinalis Extract Supplementation on Muscle Mass and Strength following Resistance Training and Detraining in Healthy Males. Sports.

[26] Seidlova-Wuttke D., Wuttke W. (2012). In a placebo-controlled study ß-Ecdysone (ECD) prevented the development of the metabolic syndrome. Planta Medica.

[27] Wuttke W., Seidlova-Wuttke D. (2012). Beta-ecdysone (Ecd) prevents visceral, bone marrow and joint fat accumulation and has positive effects on serum lipids, bone and joint cartilage. Planta Medica.

[28] World Health Organization (2000). Regional Office for the Western Pacific. The Asia-Pacific Perspective: Redefining Obesity and Its Treatment.

[29] Faul F., Erdfelder E., Lang A.-G., Buchner A. (2007). G*Power 3: A flexible statistical power analysis program for the social, behavioral, and biomedical sciences. Behav. Res. Methods.

[30] Borg G. (1970). Perceived exertion as an indicator of somatic stress. J. Rehabil. Med..

[31] Sripinyowanich S., Petchsri S., Tongyoo P., Lee T.K., Lee S., Cho W.K. (2023). Comparative Transcriptomic Analysis of Genes in the 20-Hydroxyecdysone Biosynthesis in the Fern Microsorum scolopendria towards Challenges with Foliar Application of Chitosan. Int. J. Mol. Sci..

[32] Matsuda H., Kawaba T., Yamamoto Y. (1970). Pharmacological studies of insect metamorphotic steroids. Nihon Yakurigaku Zasshi.

[33] Vierra J., Boonla O., Prasertsri P. (2022). Effects of sleep deprivation and 4-7-8 breathing control on heart rate variability, blood pressure, blood glucose, and endothelial function in healthy young adults. Physiol. Rep..

[34] Shaffer F., Ginsberg J.P. (2017). An Overview of Heart Rate Variability Metrics and Norms. Front. Public Health.

[35] Lind L., Hall J., Larsson A., Annuk M., Fellstrom B., Lithell H. (2000). Evaluation of endothelium-dependent vasodilation in the human peripheral circulation. Clin. Physiol..

[36] Graham B.L., Steenbruggen I., Miller M.R., Barjaktarevic I.Z., Cooper B.G., Hall G.L., Hallstrand T.S., Kaminsky D.A., McCarthy K., McCormack M.C. (2019). Standardization of Spirometry 2019 Update. An Official American Thoracic Society and European Respiratory Society Technical Statement. Am. J. Respir. Crit. Care Med..

[37] (2002). ATS/ERS Statement on respiratory muscle testing. Am. J. Respir. Crit. Care Med..

[38] Debouche S., Pitance L., Robert A., Liistro G., Reychler G. (2016). Reliability and Reproducibility of Chest Wall Expansion Measurement in Young Healthy Adults. J. Manipulative Physiol. Ther..

[39] Koo T.K., Li M.Y. (2016). A Guideline of Selecting and Reporting Intraclass Correlation Coefficients for Reliability Research. J. Chiropr. Med..

[40] Jalayondeja C., Jalayondeja W., Vachalathiti R., Bovonsunthonchai S., Sakulsriprasert P., Kaewkhuntee W., Bunprajun T., Upiriyasakul R. (2015). Cross-Cultural Adaptation of the Compendium of Physical Activity: Thai Translation and Content Validity. J. Med. Assoc. Thai.

[41] Baecke J.A., Burema J., Frijters J.E. (1982). A short questionnaire for the measurement of habitual physical activity in epidemiological studies. Am. J. Clin. Nutr..

[42] Cocks M., Shaw C.S., Shepherd S.O., Fisher J.P., Ranasinghe A., Barker T.A., Wagenmakers A.J. (2016). Sprint interval and moderate-intensity continuous training have equal benefits on aerobic capacity, insulin sensitivity, muscle capillarisation and endothelial eNOS/NAD(P)Hoxidase protein ratio in obese men. J. Physiol..

[43] Hasegawa N., Fujie S., Horii N., Miyamoto-Mikami E., Tsuji K., Uchida M., Hamaoka T., Tabata I., Iemitsu M. (2018). Effects of Different Exercise Modes on Arterial Stiffness and Nitric Oxide Synthesis. Med. Sci. Sports Exerc..

[44] Zador E. (2025). Molecular Targets of 20-Hydroxyecdysone in Mammals, Mechanism of Action: Is It a Calorie Restriction Mimetic and Anti-Aging Compound?. Cells.

[45] Orie N.N., Raees A., Aljaber M.Y., Mohamed-Ali N., Bensmail H., Hamza M.M., Al-Ansari N., Beotra A., Mohamed-Ali V., Almaadheed M. (2021). 20-hydroxyecdysone dilates muscle arterioles in a nitric oxide-dependent, estrogen ER-β receptor-independent manner. Phytomedicine Plus.

[46] Turnbull F. (2003). Effects of different blood-pressure-lowering regimens on major cardiovascular events: Results of prospectively-designed overviews of randomised trials. Lancet.

[47] The Blood Pressure Lowering Treatment Trialists’ Collaboration (2021). Pharmacological blood pressure lowering for primary and secondary prevention of cardiovascular disease across different levels of blood pressure: An individual participant-level data meta-analysis. Lancet.

[48] Lu Y., Wiltshire H.D., Baker J.S., Wang Q., Ying S. (2023). The effect of Tabata-style functional high-intensity interval training on cardiometabolic health and physical activity in female university students. Front. Physiol..

[49] Buniam J., Chukijrungroat N., Rattanavichit Y., Surapongchai J., Weerachayaphorn J., Bupha-Intr T., Saengsirisuwan V. (2020). 20-Hydroxyecdysone ameliorates metabolic and cardiovascular dysfunction in high-fat-high-fructose-fed ovariectomized rats. BMC Complement. Med. Ther..

[50] Tsai S.Y., Hsu J.Y., Lin C.H., Kuo Y.C., Chen C.H., Chen H.Y., Liu S.J., Chien K.L. (2024). Association of stress hormones and the risk of cardiovascular diseases systematic review and meta-analysis. Int. J. Cardiol. Cardiovasc. Risk Prev..

[51] Songsorn P., Somnarin K., Jaitan S., Kupradit A. (2022). The effect of whole-body high-intensity interval training on heart rate variability in insufficiently active adults. J. Exerc. Sci. Fit..

[52] Heydari M., Boutcher Y.N., Boutcher S.H. (2013). High-intensity intermittent exercise and cardiovascular and autonomic function. Clin. Auton. Res..

[53] Phungphong S., Kijtawornrat A., Chaiduang S., Saengsirisuwan V., Bupha-Intr T. (2017). 20-Hydroxyecdysone attenuates cardiac remodeling in spontaneously hypertensive rats. Steroids.

[54] Zaroni R.S., Brigatto F.A., Schoenfeld B.J., Braz T.V., Benvenutti J.C., Germano M.D., Marchetti P.H., Aoki M.S., Lopes C.R. (2019). High Resistance-Training Frequency Enhances Muscle Thickness in Resistance-Trained Men. J. Strength Cond. Res..

[55] Heydari M., Freund J., Boutcher S.H. (2012). The effect of high-intensity intermittent exercise on body composition of overweight young males. J. Obes..

[56] Sieck G.C., Ferreira L.F., Reid M.B., Mantilla C.B. (2013). Mechanical properties of respiratory muscles. Compr. Physiol..

[57] Dioh W., Chabane M., Tourette C., Azbekyan A., Morelot-Panzini C., Hajjar L.A., Lins M., Nair G.B., Whitehouse T., Mariani J. (2021). Testing the efficacy and safety of BIO101, for the prevention of respiratory deterioration, in patients with COVID-19 pneumonia (COVA study): A structured summary of a study protocol for a randomised controlled trial. Trials.

[58] Burgomaster K.A., Howarth K.R., Phillips S.M., Rakobowchuk M., Macdonald M.J., McGee S.L., Gibala M.J. (2008). Similar metabolic adaptations during exercise after low volume sprint interval and traditional endurance training in humans. J. Physiol..

[59] Zhang X., Yang L., Xiao C., Li J., Hu T., Li L. (2024). Association between waist-to-hip ratio and risk of myocardial infarction: A systematic evaluation and meta-analysis. Front. Cardiovasc. Med..

[60] Robledo-Millan C.R., Diaz-Dominguez M.R., Castaneda-Ramirez A.E., Quinones-Lara E., Valencia-Marin S., Suarez-Garcia R.X., Lopez-Desiderio N.G., Ramos-Cortes C.A., Gaytan Gomez A.M., Bello-Lopez J.M. (2025). A Novel Metabolic Risk Classification System Incorporating Body Fat, Waist Circumference, and Muscle Strength. J. Funct. Morphol. Kinesiol..

[61] Liu J., Xu H., Cupples L.A., GT O.C., Liu C.T. (2023). The impact of obesity on lung function measurements and respiratory disease: A Mendelian randomization study. Ann. Hum. Genet..

